# The Direct Interaction between Two Morphogenetic Proteins Is Essential for Spore Coat Formation in *Bacillus subtilis*

**DOI:** 10.1371/journal.pone.0141040

**Published:** 2015-10-20

**Authors:** Rachele Isticato, Teja Sirec, Stefano Vecchione, Anna Crispino, Anella Saggese, Loredana Baccigalupi, Eugenio Notomista, Adam Driks, Ezio Ricca

**Affiliations:** 1 Department of Biology, Federico II University, Naples, Italy; 2 Department of Microbiology and Immunology, Infectious Disease and Immunology Research Institute, Loyola University Chicago, Maywood, IL, United States of America; Institut Pasteur Paris, FRANCE

## Abstract

In *Bacillus subtilis* the protective layers that surround the mature spore are formed by over seventy different proteins. Some of those proteins have a regulatory role on the assembly of other coat proteins and are referred to as morphogenetic factors. CotE is a major morphogenetic factor, known to form a ring around the forming spore and organize the deposition of the outer surface layers. CotH is a CotE-dependent protein known to control the assembly of at least nine other coat proteins. We report that CotH also controls the assembly of CotE and that this mutual dependency is due to a direct interaction between the two proteins. The C-terminal end of CotE is essential for this direct interaction and CotH cannot bind to mutant CotE deleted of six or nine C-terminal amino acids. However, addition of a negatively charged amino acid to those deleted versions of CotE rescues the interaction.

## Introduction

Many biological systems depend on molecular self-assembly to create complex supramolecular structures that carry out diverse functions. Those complex structures are generally based on noncovalent interactions between the forming molecules and require strict regulatory mechanisms. The spore coat of the gram-positive bacterium *Bacillus subtilis* is an example of such supramolecular structure and, because of the amenability of this microorganism to genetic and molecular analysis, is a model to study and improve our knowledge on the formation of self-assembled structures.

Spore formation starts when cell growth is no longer allowed by nutrient starvation or other environmental conditions affecting DNA replication [1, 2]. First morphological step of spore formation is an asymmetric cell division that produces a large mother cell and a small forespore. The mother cell contributes to forespore maturation and undergoes autolysis at the end of the process allowing the release of the mature spore into the environment [1, 2]. The mature spore is extremely stable and resistant to harsh conditions. It can survive for extended periods of time in the absence of water and nutrients and to extremes of heat and pH, to UV radiation, and to the presence of solvents, hydrogen peroxide and lytic enzymes [3, 4]. The spore is, however, able to sense the environment and respond to the presence of water and nutrients generating a cell able to grow and, eventually, to re-sporulate [5].

The resistance properties of the spore are due to its unusual structure and physiology. The dehydrated cytoplasm, containing a copy of the chromosome, is surrounded by a series of protective layers. A peptidoglycan-like cortex is the first shield, surrounded by a multilayered coat and a crust [6]. Coat and crust together are composed of at least 70 different proteins and glycoproteins. While several proteins of both coat and crust have been identified and characterized, little is known about which of them is glycosylated and the identities of the sugars on the spore surface. It is, however, known that the presence of glycoproteins on the spore surface modulates the relative hydrophobicity of the spore [7, 8]. Coat formation is finely controlled by a variety of mechanisms acting at various levels. The synthesis of coat proteins is regulated by at least two mother cell-specific sigma subunits of RNA polymerase and at least three additional transcriptional regulators. These transcription factors act in a temporal sequence, and differentially in the forespore and mother cell, thereby controlling the time and cell type of expression of the coat structural genes (*cot* genes) [6]. At least some coat proteins are subject to post-translation maturation events, including proteolytic cleavage, cross-linking, phosphorylation and glycosylation reactions [9]. An important subset of coat proteins, referred to as morphogenetic coat proteins, have especially important roles in coat formation, in that these proteins direct the assembly of others in the coat, forming a complex network of interactions [10–14]. Within this subset of regulatory coat proteins, CotE plays a crucial role in the assembly of outer coat and crust: without it, these layers are not assembled [15]. Consistent with this role, CotE is present between the inner and outer coat layers in the mature spore [16]. Interestingly, CotE is also found in the mother cell cytoplasm, up to at least eight hours after the start of sporulation [17]. A mutagenesis study showed that CotE has a modular structure: a C terminal domain that directs the assembly of various coat proteins including some known to be in the outer coat layer, an internal domain involved in targeting CotE to the forespore and a N terminal domain that, together with the internal domain, directs the formation of CotE multimers [18, 19]. More recently, formation of CotE multimers was confirmed by yeast-two-hybrid analysis [20]. A global study of the coat protein interaction network in *B*. *subtilis* suggested that CotE interacts, directly or indirectly, with most outer coat proteins [10, 12]. A direct interaction with CotE has been demonstrated for SpoVID [14], CotC and CotU [21]. In the case of CotC and CotU, the interaction with CotE is essential for CotC-CotU heterodimerization [21].

CotH is a CotE-dependent (i.e., CotH assembly depends on CotE) [22, 23] morphogenetic protein responsible of the assembly of at least 9 other coat proteins, including CotG, CotC, CotU and CotS [12]. In the absence of CotH, the coat is severely altered and spore resistance and germination is severely impaired [22, 23] At least in part, these phenotypes are likely the result of the lack of an important subset of outer coat proteins [10, 22]. CotH assembly can be engineered to be CotE independent, by over-expression of the *cotH* gene [24]. This suggests that CotE facilitates CotH assembly but is not necessarily essential for this event. There is also evidence that at least some degree of CotE assembly is CotH-dependent [12], suggesting a role for CotH in stabilizing CotE at the spore surface after the initial deposition of CotE. Mutant spores lacking both CotH and CotE germinate less efficiently and showed an increased sensitivity to lysozyme than single *cotE* mutant spores [22]. This suggests additional roles for CotH that are CotE-independent. Taken as a whole, these prior studies establish that CotH plays a major role in coat assembly and, as a result, in key spore properties. However, they leave unclear the mechanism(s) by which CotE directs CotH assembly into the coat. In particular, it remains unknown whether additional coat proteins mediate this pivotal interaction.

In the present work, we focus on the CotE-CotH interaction and on its role in coat formation. We further characterize the dependency of CotE assembly on CotH, and provide evidence arguing that the CotE-CotH interaction is independent of any other morphogenetic coat proteins. Based on our results, we propose a revised model for the assembly of the CotH-dependent portion of the outer coat.

## Materials and Methods

### General methods, analysis of spore proteins and immunoassays

Strains and primers used for polymerase chain reaction (PCR) are listed in S1 and S2 Tables, respectively. Manipulations of *B*. *subtilis* were performed as described previously [25]. Sporulation was induced by the by exhaustion method in Difco Sporulation Medium (DSM). Ten milliliters of sporulating cells were harvested at various times during sporulation and mother cells and forespore fractions isolated as described before [26]. Whole-cell lysates of sporulating cells were prepared by sonication [26] followed by detergent treatment (62.5 mM Tris-HCl (pH 6.8), 4% SDS, 5% glycerol, 2% beta-mercaptoethanol, 0.003% bromophenol blue) at 100°C for 7 min. 50 μg (mother cell extract or whole-cell lysates) or 20 μg (forespore extract) of total proteins was used for western blot analysis. Extraction of proteins from mature spores (from fifteen milliliters culture) was performed with treatment at 65°C in SDS-DTT extraction buffer or at 4°C in 0.1 M NaOH [24]. Western blot analysis were performed by standard procedures. For electrotransfer was used nitrocellulose membrane and the proteins were then hybridized with either anti-CotH, anti- CotE anti-CotB, anti-CotG or anti-CotC antibodies as described previously [26].

### Strain construction and recombinant DNA procedure

Recombinant DNA procedures were carried out as described previously [27] unless otherwise indicated. CotE mutants were obtained by amplifying the *cotE* gene by PCR using the *B*. *subtilis* chromosomal DNA as a template and the CotE-P synthetic oligonucleotide (S2 Table) as forward primer. Four different oligonucleotides, CotE 525-9EE-R, CotE 525-6D-R, CotE 525-6E-R, CotE 525-6K-R (S2 Table) were independently used as reverse primers. All amplification fragments (all of about 920 bp) were firstly cloned in pGem-T (*Promega*) and then excised by enzymatic digestion using *BamH*I and *EcoR*I restriction enzymes and finally cloned into pDG364 vector, previously digested with the same enzymes. By this strategy plasmids pTS25 (9EE), pTS22 (6D), pTS23 (6E) and pTS24 (6K) were obtained. All four plasmids were sequenced and then used to transform a *B*. *subtilis cotE* null mutant strain (RH211), obtaining RH401 (-9EE), RH402 (-6D), RH403 (-6E) and RH404 (-6K) mutant strains.

### Overproduction of proteins in *E*. *coli* and pull-down experiments

*cotH* coding region was PCR amplified using the *B*. *subtilis* chromosomal DNA as a template and oligonucleotides H34 and H35 (S2 Table) to prime the reaction. The obtained fragment of 1,100 bp was digested *SacI* and *KpnI* restriction enzymes and cloned in frame with poly-His tag into the pBAD-B expression vector (*Life Technologies*) previously digested with the same enzymes. To over-produce CotE -9EE, -6D or -6E, the *cotE* coding regions were amplified by PCR using the oligonucleotides E-NdeI-F and pDG364 720R-HindIII (S2 Table) to prime the reactions and chromosomal DNA of strains RH401, RH402 or RH403 as templates. All fragments were digested with *Nde*I and *Hind*III restriction enzymes and then cloned the pRSET-B expression vector (*Invitrogen*) previously digested with the same enzymes. All the resulting plasmids were checked by nucleotide sequence analysis and used to transform *E*. *coli* strain BL21(DE3) (Novagen) to create the strains RH406 (-9EE), RH405 (-6D) and RH407 (-6E) (S1 Table).

For the His tag pull down assays, CotH-His and all the untagged modified versions of CotE were produced using the *Overnight Express Autoinduction Media* as previoulsy described [21]. Briefly, after an over-night incubation at 37°C in 15 mL of autoinduction medium (*Novagen*), the cells of all strains were collected by centrifugation and resuspended in 1.5 ml of lysis buffer (50 mM NaH_2_PO4, 300 mM NaCl, 10 mM imidazole, 2 mg/ml lysozyme, and 0.01 mg/ml RNase). After 30 min at 4°C, the lysates were sonicated and the suspension was clarified by centrifugation at 13,000 g at 4°C for 20 min. 300μg of extract from strain expressing CotH-His was applied to Ni-NTA magnetic agarose beads (*Qiagen*). After 1 h of incubation at room temperature with shaking, the beads were washed with 2.5 ml of wash buffer (50 mM NaH_2_PO4, 300 mM NaCl, 10 mM imidazole), and 300 μg of extract from strain RH134 (or RH404, or RH405 or RH406) was added to the beads and incubated for 1 h at room temperature with shaking to facilitate binding. Unbound proteins were removed by washing with wash buffer at three different concentrations of imidazole (40 mM, 100 mM, and 250 mM. Bound proteins were eluted using the wash buffer at increasing concentrations of imidazole (500 mM and 1 M) Eluted proteins were resolved on 12.5% SDS-PAGE gels and subjected to immunoblot analysis [26].

## Results and Discussion

### CotE assembly depends on CotH

**Fig 1:**
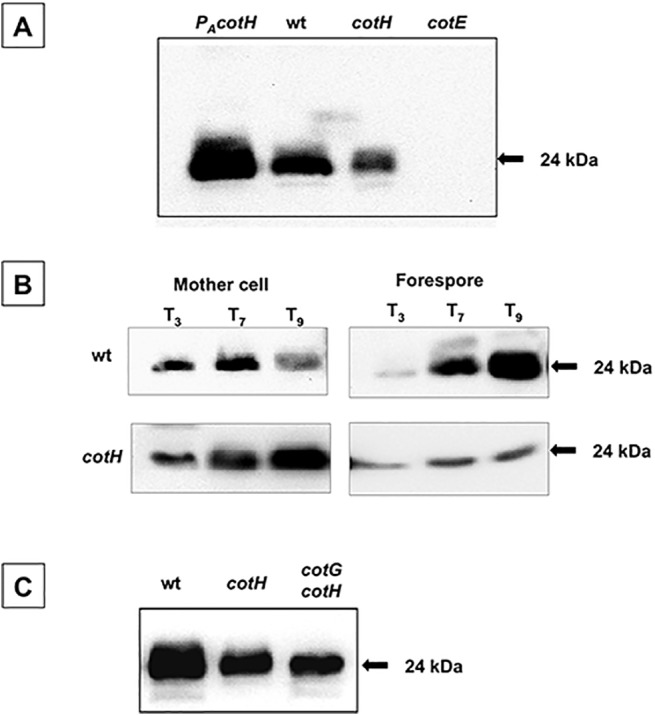
CotE *in vivo* assembly in mature spores and during sporulation. Proteins for western blot analysis were extracted from: (A) mature spores of a wild type strain (wt), or isogenic strains lacking CotH (*cotH;* ER220) or CotE (*cotE;* RH211) or over-producing CotH (*P*_*A*_ *cotH;* RG24) (24); (B) mother cell or forespore compartments of sporulating cells of a wild type strain (wt), or an isogenic strain lacking CotH (*cotH;* ER220), 3 (T_3_), 7 (T_7_), and 9 (T_9_) hours after the initiation of sporulation; (C) mature spores of a wild type strain (wt), or isogenic strains lacking CotH (*cotH;* ER220), or CotG and CotH (*cotG cotH;* AZ603). Proteins were fractionated on 15% SDS-PAGE, electrotransfered on a membrane and reacted with anti-CotE antibody.

A western blot approach with anti-CotE-antibody was used to investigate the degree to which CotE coat assembly within the coat is dependent on CotH. Fig 1A shows that the amount of extracted CotE is higher in mature spores of a wild type *B*. *subtilis* strain than in spores of a congenic *cotH* mutant strain (strain ER220). Consistent with the possibility that CotE assembly is due, at least in part, to direct interactions with CotH, we found that the amount of extracted CotE is higher in spores engineered to over express *cotH* (strain RG24) than in wild type spores (Fig 1A). We conclude from these experiments that the assembly of CotE in mature spores depends on the level of CotH.

To more fully characterize the consequences of CotH on CotE assembly, we measured the levels of CotE in each of the two compartments of sporulating cells. To do this, we applied western blot analysis with anti-CotE-antibody on proteins extracted from either the mother cell or the forespore compartment at various times after the onset of sporulation. As previously reported [17], in a wild type strain, three hours after the onset of sporulation (T_3_), CotE was mostly found in the mother cell compartment (Fig 1B). After that it was found in both compartments (T_7_) and, somewhat later (T_9_), CotE was mostly in the forespore. In a congenic strain lacking CotH, at all time points tested the majority of CotE was found in the mother cell cytoplasm (Fig 1B). These results suggest that CotH acts at the level of CotE incorporation into the coat, and not at the level of CotE synthesis or stability. These data provide strong confirmation of previous results suggesting that CotE is a CotH-controlled protein [12].

Recent work suggests that CotH counteracts a negative role played by CotG [28]. In that study, it was shown that the assembly of at least three coat proteins, CotC, CotU and CotS, requires CotH only when CotG is present. Indeed, although all three proteins fail to assemble in spores of a strain lacking CotH, the defect is eliminated when CotG is also lacking. Therefore, we addressed the hypothesis that CotG contributes to CotH-dependent CotE assembly, by measuring CotE assembly in a strain lacking both CotH and CotG (strain AZ603). As shown in Fig 1C, the amount of CotE assembled around mature spores of a strain lacking only CotH or both CotH and CotG is similar, demonstrating that CotE assembly is not negatively affected by CotG.

### CotE and CotH interactions *in vitro*

**Fig 2:**
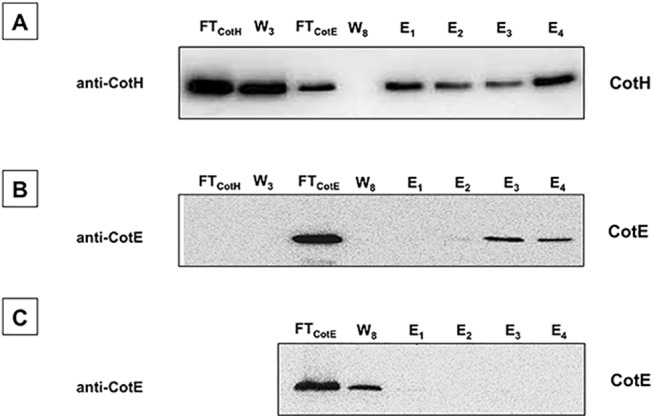
Immunoprecipitation analysis of the *in vitro* CotH-CotE interactions. CotH-His was bound to a Ni-NTA column and the flowthrough (FT_CotH_) and washes (W1-W3, here only W3 is shown) were collected. Untagged CotE was then added, and flow through (FT_CotE_), washed (W1—W8, here only W8 is shown), and eluted (E1—E4) proteins collected as described in Materials and Methods. Proteins were fractionated on 12.5% polyacrylamide gels, electrotransferred to membranes, and reacted with anti-CotH (A), anti-CotE (B) antibodies. The same experiment was also performed without CotH-His (C).

To address the key question of whether any *B*. *subtilis* coat proteins mediate the interaction between CotE and CotH, we overproduced in *E*. *coli* a His-tagged version of CotH (CotH-His) and an untagged version of CotE, and performed an *in vitro* His tag pull-down assay. To do this, we lysed *E*. *coli* cells producing CotH-His, incubated them with Ni-NTA magnetic beads and then incubated the beads with an extract of cells producing untagged CotE. We then eluted proteins from the beads, fractionated them by SDS-PAGE and performed blot analysis, with anti-CotH (Fig 2A), anti-CotE (Fig 2B and 2C) or anti-His (not shown) antibodies. A small fraction of untagged CotE was able to bind Ni-NTA beads when CotH-His was present (Fig 2B). In the absence of CotH-His, untagged CotE was not able to bind to the Ni-NTA beads (Fig 2C). These data argue against the view that a *B*. *subtilis* coat protein is required for the CotE-CotH interaction. The simplest interpretation of these data is that the interaction between these two proteins is direct.

### The C terminus of CotE protein is involved in the interaction with CotH

**Fig 3:**
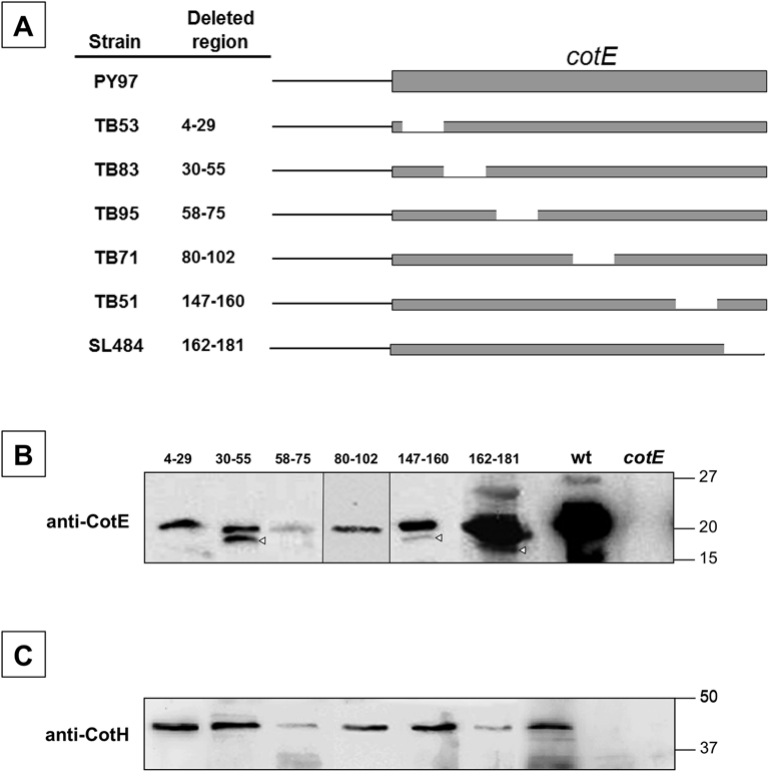
Effects of deletions in CotE on *in vivo* CotH assembly. (A) CotE and various deletion mutant versions of the protein are indicated. To the left of each construct is the strain name and the deleted amino acids. Coat proteins were extracted from mature spores of wild type and mutants, and analyzed by western blot with anti-CotE (B) or anti-CotH (C) antibodies. White triangles indicate likely CotE degradation products. Molecular masses are indicated in kilodaltons.

Previous studies demonstrated that the CotE sequence is organized into functional modules, where the N- and C-termini have roles in the assembly of the outer coat proteins, and an internal block of residues directs the assembly of CotE to the developing forespore [18, 19]. To identify regions of CotE that mediate interactions with CotH, we analyzed a collection of congenic *cotE* mutants (Fig 3A; [17]), each harboring a different 20 amino acid deletion in the resulting protein. Western blot analysis showed that CotE was present in spores from all the mutant strains (Fig 3B). As expected [18], the levels of CotE present in the various strains is lower than in wild type strain. Also as expected, CotE was only barely detectable in spores of strain TB95, due to the functions of the missing amino acids (58 to 75) (Fig 3B) [18]. Western blot analysis with anti-CotH antibodies demonstrated the presence of similar levels of CotH in spores of all the mutant strains except TB95 (presumably because of the low level of CotE) and in strain SL484 (missing amino acids 162–181) (Fig 3C). We conclude, therefore, that amino acids within 162–181 participate in CotH assembly. This finding is consistent with previous work, showing that that the absence of these residues results in low levels of assembly of the CotH-dependent protein CotG [19]. Our finding is also consistent with the view that the interaction between CotE and CotH in the coat is direct.

### Role of a negatively charged amino acid at the C terminus of CotE

**Fig 4:**
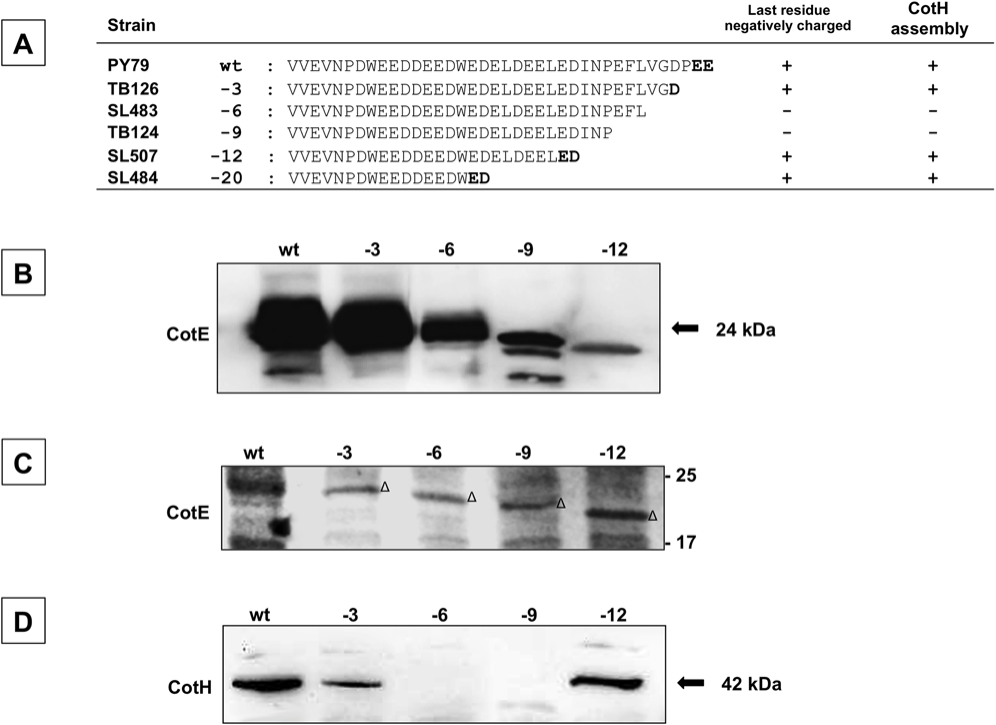
Effects of C-terminal deletions of CotE on *in vivo* CotH assembly. (A) The amino acid sequences of the C termini of CotE and various mutant versions. To the left of each sequence are the strain name and the number of deleted amino acids. Negatively charged amino acids when present at the C terminus are indicated in bold. Western blot with anti-CotE antibody (B), SDS-PAGE (C) and western blot with anti-CotH- antibody (D) of coat proteins extracted from spores of the strain indicated in panel A. Triangles in panel C indicate the predicted CotE bands. Proteins were fractionated on 12.5% polyacrylamide gels, electrotransferred to membranes, and reacted with anti-CotH and anti-CotE antibodies.

To identify more precisely the amino acids in CotE that direct CotH assembly, we analyzed CotH assembly in spores of strains TB126, SL483, TB124 and SL507 lacking the last 3, 6, 9 or 12 residues at the C terminus of CotE, respectively [19] (Fig 4A). Western blot with anti-CotE antibody and SDS-PAGE analyses showed that in spores of all mutant strains, CotE was present, and at levels consistent with those reported previously (Fig 4B and 4C) [19]. Western blot analysis with anti-CotH antibody revealed that CotH was present when the 3 C terminal-most amino acids were missing (in strain TB126) but was absent when the 6 or 9 C terminal-most amino acids were missing (in strains SL483 and TB124) (Fig 4D). Interestingly, CotH was detected when twelve (strain SL507 in Fig 4D) or twenty (strain SL484 in Fig 3) amino acids were deleted. These data are striking in light of previous data indicating that the deletion of 6 or 9 amino acids did not prevent assembly of the CotH-dependent proteins, including CotG [19].

**Fig 5:**
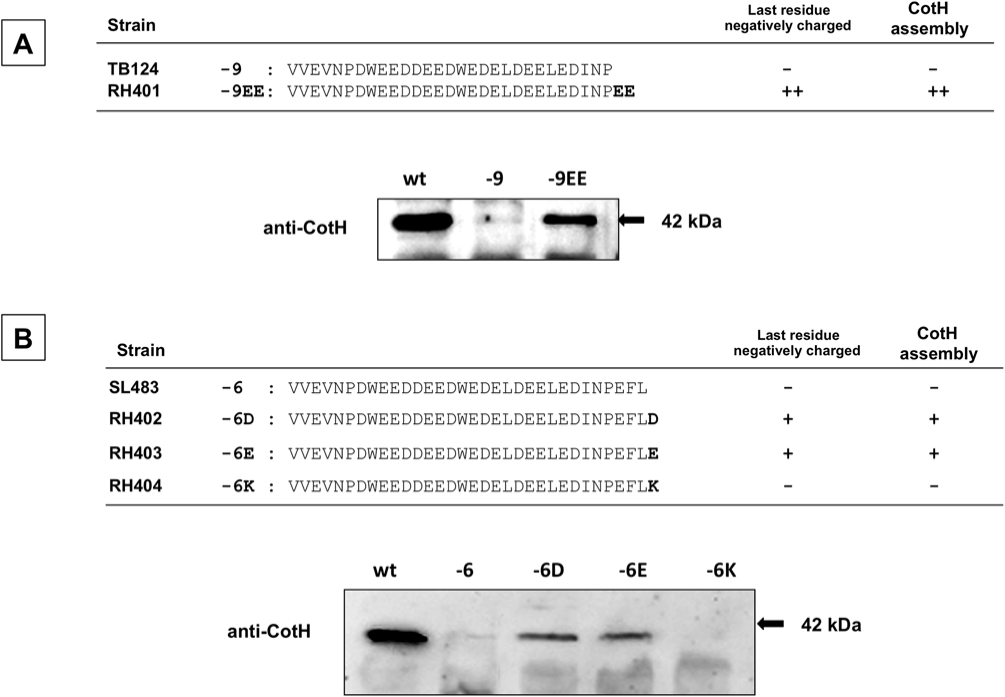
Rescue of *in vivo* CotH assembly by addition of negatively charged residues. Strains bearing versions of CotE lacking nine (A) or six (B) C-terminal amino acids were modified by adding the indicated C terminal residue and analyzed by western blot. Proteins were fractionated on 12.5% polyacrylamide gels, electrotransferred to membranes, and reacted with anti-CotH antibody.

While seeking an explanation of the data just described, we noted that in all the versions of CotE that successfully assembled CotH (wild type, and those missing 3, 12 or 20 C terminal amino acids), but not the versions that failed to assemble CotH, the final residue was negatively charged (Fig 4A). To test the hypothesis that to direct CotH assembly CotE must have a negatively charged C terminus, we generated a strain bearing a version of CotE lacking the C-terminal 9 amino acids and with the addition of two glutamic acid (EE) residues (strain RH401). Western blot analysis showed that spores from this strain do assemble CotH (Fig 5A). To further address our hypothesis, we generated strains bearing versions of CotE lacking the 6 C-terminal residues and with the addition of either an aspartic acid, a glutamic acid or a lysine (strains RH402, RH403 and RH404, respectively) (Fig 5B). Western blot analysis demonstrated that CotH assembly was restored by the addition of the negatively, but not the positively charged amino acids (Fig 5B).

**Fig 6:**
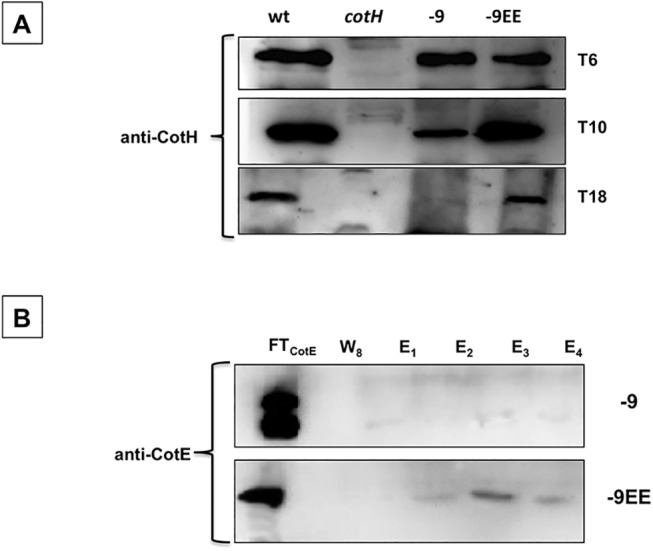
Time course of *in vivo* CotE-CotH interaction and *in vitro* pull down experiments. (A) Western blot of proteins extracted from the forespore compartment of sporulating cells of a wild type strain (wt), or isogenic strains lacking CotH (*cotH;* ER220), carrying a deleted version of CotE (-9; TB124) or carrying a deleted version with a negatively charged C terminus (-9EE). Proteins were extracted 6 (T_6_), 10 (T_10_), and 18 (T_18_) hours after the initiation of sporulation. (B) Untagged CotE -9 and -9EE versions were independently added to a Ni-NTA column bound to CotH-His as in Fig 2A. Flow through (FT_CotE_), washed (W1—W8, here only W8 is shown), and eluted (E1—E4) proteins collected as described in Materials and Methods. Proteins were fractionated on 12.5% polyacrylamide gels, electrotransferred to membranes, and reacted with anti-CotH and anti-CotE antibodies.

Previous experiments showed that CotH assembly to the spore becomes strictly dependent on CotE only at late times during sporulation (after T11) (24). Therefore, we asked whether the presence of a positive amino acid to the CotE C terminus (strain TB124 lacking 9 C-terminal residues) would affect this early phase of apparently CotE-independent CotH assembly. We found that the pattern of CotH assembly was similar to that previously seen in a *cotE* null mutant strain (strain RG25 in [24]); CotH was found around the spore at early times during sporulation (T6 and T10) but not at a late time (T18) (Fig 6A) or in mature spores (Fig 5). When a negatively charged amino acid was added a wild type pattern of CotH assembly was rescued (Fig 6A). Therefore, the presence of a positive residue to the CotE C-terminus does not prevent early CotH assembly.

We then conclude that during sporulation CotH initially assembles around the spore in a CotE-independent way and that the interaction with CotE is essential to stabilize CotH presence around the forming spore. Such interaction strictly requires the presence of a negatively charged amino acid as C terminus of CotE.

### CotH interactions *in vitro* with CotE missing 9 amino acids but with an additional negatively charged residue

Our studies so far suggested the possibility that a negatively charged amino acid on the C terminus of CotE interacts directly with CotH. If so, then we would expect an appropriately engineered version of CotE to interact with CotH in the *in vitro* assay already described. To address this, we analyzed interactions between CotH and overproduced versions of CotE with deletions of 6 or 9 amino acids, and with or without the addition of an acidic residue. As shown in Fig 6B, CotE lacking 9 amino acids was unable to pull down CotH-His, but was able to when a negatively charged residue was added to the C terminus. The same result was obtained using the version of CotE lacking the 6 C-terminal residues: this version of CotE did not pull down CotH-His, but the pull down was successful when a glutamic acid (E) residue was added (not shown).

### The interaction of CotH with a truncated version of CotE is functional

**Fig 7:**
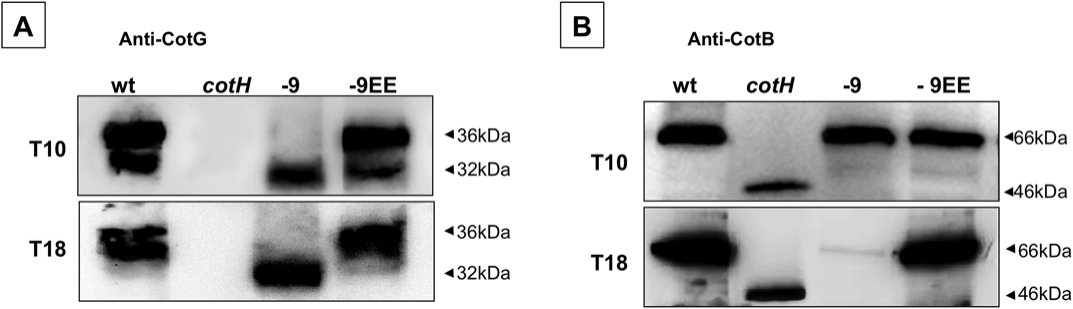
Effects of CotE mutations on CotB and CotG assembly during sporulation. Western blot with anti-CotG (A) and anti-CotB (B) antibodies of proteins extracted from mature spores of a wild type strain (wt) or of isogenic strains lacking CotH (*cotH*; ER220) or carrying modified version of CotE (-9; TB124, -9EE; RH401). Proteins were fractionated on 12.5% polyacrylamide gels, electrotransferred to membranes, and reacted with the anti-CotG (A) and anti-CotB (B) antibodies.

In the experiment just described, we showed that addition of a negatively charged amino acid to a version of CotE missing either the C-terminal -6 and -9 amino acids rescued CotH assembly. To further characterize the degree of rescue of CotH function by these versions of CotE, we next tested whether they directed assembly of the CotH-controlled proteins CotB, CotC, CotG, and CotU. CotG is an abundant protein extracted from wild type spores in a 32 and a 36 kDa form both strictly dependent on CotH for coat assembly [12, 28, 29] (Fig 7A). The removal of the C-terminal 6 and 9 amino acids from CotE results in the loss of the 36 kD but not the 32 kD CotG species (Fig 7A and data not shown). The addition of a negatively charged residue to the C terminus of the truncation restored the presence of the 36 kDa species (Fig 7A and data not shown). The simplest interpretation of these results is that assembly of the 36 kDa species depends on an interaction between CotE and CotH. Our data also argue that assembly of the 32 kDa species requires CotH but it does not depend on a CotE-CotH interaction, implying that CotH can participate in assembly of CotG-32 even if it is in the mother cell cytoplasm and not bound into the coat (Fig 6A).

CotB is extracted from wild type spores in its 66 kDa mature form [30]. However, CotB is produced as a 46 kDa species whose maturation requires CotH and CotG [30]. Prior to the point in coat assembly when CotH deposition is independent of CotE (T10) (Fig 6A), the mature, 66 kDA form of CotB is present in the spore of a strain containing either the 6 or 9 amino acid truncation version of CotE, (Fig 7B and data not shown). In the same strain but at a later time (T18), CotH is no longer assembled within the coat (Fig 6A and data not shown) and also the mature form of CotB is no longer present in the coat (Fig 7B and data not shown). The presence of a negatively charged amino acids to either CotE truncation construct rescues CotH assembly and also assembly of the mature form of CotB (Fig 7B and data not shown).

**Fig 8:**
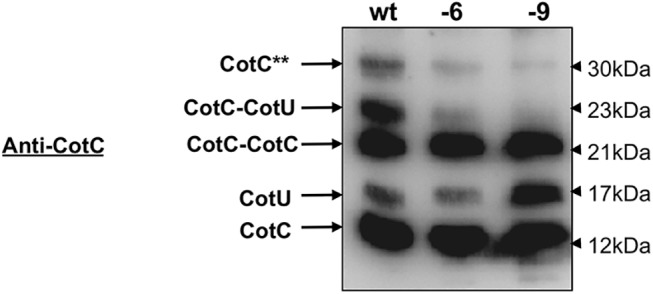
Effects of CotE mutations on assembly of CotC and CotU during sporulation. Western blot with anti-CotC antibody of proteins extracted from mature spores of a wild type strain (wt), or isogenic strains carrying either deleted version of CotE (-6; SL483, -9; TB124). The various forms of CotC and CotU are indicated. CotC** indicates a post-translationally modified form of CotC [31]. Proteins were fractionated on 12.5% polyacrylamide gels, electrotransferred to membranes, and reacted with the indicated antibodies.

CotC and CotU share significant sequence identity and are both recognized by anti-CotC and anti-CotU antibodies [31, 32]. They are assembled in several forms including a CotC-CotU heterodimer of 23 kDa whose formation requires a direct interaction with CotE [21]. CotH plays a role in this event, by counteracting a still poorly understood negative role played by CotG on CotC and CotU assembly [28]. Assembly of CotU and CotC monomer forms, and CotC dimers, was not significantly affected by deletion of the CotE C terminus (Fig 8A). However, we did detect significant changes in the levels of the 23 kDa CotC-CotU heterodimer and the 30 kDa form of CotC [31, 32], that appear both to be strongly reduced in spores bearing either CotE truncation construct (Fig 8A). Assembly of these species were not rescued by the addition of a negatively charged amino acid (data not shown).

## Conclusions

The assembly of CotH and CotE is mutually dependent. CotH dependence on CotE has been established in several previous studies [23, 24, 30].The dependence of CotE on CotH is much less well characterized [12]. Here we further characterize this interaction and use the results to generate a refined model for coat assembly. Our principal finding is that both the CotE-dependent assembly of CotH, and the CotH-dependent assembly of CotE, require amino acids at the CotE C-terminus, and the effects of removal of these residues is overridden by the addition of a negatively charged amino acid to the end of the construct. We suggest that the simplest interpretation of these data is that the mutually dependent assembly of CotE and CotH is due to a direct interaction between these proteins. CotH has at least two roles. First, it directs the assembly of a large subset of coat proteins [12]. Second, CotH stabilizes the already deposited CotE.

Our results also provide insights into the mechanism by which CotG negatively effects assembly of CotC and CotU [28]. Our data suggest a model in which the 32 kDa form of CotG (whose assembly is CotH-independent) does not have the ability to exert the negative affect on CotC or CotU assembly (that CotG usually produces in *cotH* mutant spores [28]), but the 36 kDa form does. This result is striking because it is, to our knowledge, the first evidence for a difference in function in assembly between isoforms of a coat protein. We suggest, therefore, that in addition to the functions already described for CotH, a key role is, directly or indirectly, to suppress a deleterious effect of the 36 kDa form of CotG on coat assembly. This possible role underscores what is likely to be an important general feature of coat protein assembly; the need to suppress certain possible interactions which, left unchecked, lead to misassembled coats. We speculate that there are other interactions between coat proteins whose major or even primary function is to prevent maladaptive assembly. Possibly, the potential for deleterious interactions among a relatively large number of coat proteins has led to a correspondingly large number of such "prophylactic" interactions and, therefore, a surprisingly high degree of biochemical complexity to the coat as a whole.

## Supporting Information

S1 Table List of strains used in this study. (DOCX)

S2 Table List of primers. (DOCX)
