# Supplementary material for: The Direct Interaction between Two Morphogenetic Proteins Is Essential for Spore Coat Formation in Bacillus subtilis
Source: PLoS One. 2015 Oct 20;10(10):e0141040. doi: 10.1371/journal.pone.0141040 (PMC4618286; doi:10.1371/journal.pone.0141040)
Supplement: S1 Table — (DOCX) [file pone.0141040.s001.docx]

**S1 Table.** List of strains used in this study.

| Strain | Genotype | Source^a^ | |  |
| --- | --- | --- | --- | --- |
| *Bacillus subtilis* |  |  | |  |
| PY79 | wild type | | reference below | |
| RH211 | *cotE::spc* | [21] | |  |
| ER220 | *cotH::spc* | [22] | |  |
| RG24  AZ603 | *pAH::cat*  *ΔcotG ΔcotH::neo* | [24]  [25] | |  |
| TB70 | *cotE::cat::spc amyE::cotEΔ159-181* | [18] | |  |
| TB51 | *cotE::cat::spc amyE::cotEΔ147-160* | [18] | |  |
| TB71 | *cotE::cat::spc amyE::cotEΔ80-102* | [18] | |  |
| TB95 | *cotE::cat::spc amyE::cotEΔ58-75* | [18] | |  |
| TB83 | *cotE::cat::spc amyE::cotEΔ30-55* | [18] | |  |
| TB126 (-3) | *cotE::cat::spc amyE::cotEΔ179–181* | [19] | |  |
| SL483 (-6)  TB124 (-9) | *cotE::cat::spc amyE::cotEΔ176–181*  cotE::*cat::spc amyE::cotEΔ173–181* | [19]  [19] | |  |
| SL507 (-12) | *cotE::cat::spc amyE::cotEΔ170–181* | [19] | |  |
| SL484 (-20) | *cotE::cat::spc amyE::cotEΔ162–181* | [19] | |  |
| RH401 (-9EE) | *cotE::spc::cat* amyE::*cotEΔ173–181EE* | This study | |  |
| RH402 (-6D) | *cotE::spc::cat* amyE:: *cotEΔ176–181D* | This study | |  |
| RH403 (-6E) | *cotE::spc::cat* amyE::*cotEΔ176–181E* | This study | |  |
| RH404 (-6K) | *cotE::spc::cat* amyE::*cotEΔ176–181K* | This study | |  |
| *Escherichia coli* ^b^ |  |  | |  |
| CotH-His | *cotH::6his* | This study | |  |
| RH134 | *cotE* | [21] | |  |
| RH405 | *cotEΔ173–181EE* | This study | |  |
| RH406 | *cotEΔ176–181D* | This study | |  |
| RH407 | *cotEΔ176–181E* | This study | |  |

^a^ Numbers refer to references in the text. ^b^ All *E. coli* strains are derivatives of strain BL21(DE3) transformed with various plasmids. The relevant genotypes shown for *E. coli* strains are those of the contained plasmid.

Reference:

- Youngman P, Perkins JB, Losick R. A novel method for the rapid cloning in *Escherichia coli* of *Bacillus subtilis* chromosomal DNA adjacent to Tn917 insertion. Mol. Gen. Genet. 1984; 195:424-433.
