# Supplementary material for: The Direct Interaction between Two Morphogenetic Proteins Is Essential for Spore Coat Formation in Bacillus subtilis
Source: PLoS One. 2015 Oct 20;10(10):e0141040. doi: 10.1371/journal.pone.0141040 (PMC4618286; doi:10.1371/journal.pone.0141040)
Supplement: S2 Table — (DOCX) [file pone.0141040.s002.docx]

**S2 Table.** List of primers

| Oligonucleotide | Sequence^a, b^ | Restriction site | | Position of annealing^c^ | |
| --- | --- | --- | --- | --- | --- |
| CotE-P | CGggatccCGAGCTCGTTGCACACACC | | BamHI | -370/-388 (*cotE*) |  |
| CotE 525-9EE-R | aagcttATTCCTCCGGGTTGATGC | | HindIII | +505/+519(*cotE*) |  |
| CotE 525-6D-R | aagcttAGTCTAAAAACTCCGGGTTG | | HindIII | +510/+526(*cotE*) |  |
| CotE 525-6E-R | aagcttATTCTAAAAACTCCGGGTTG | | HindIII | +510/+526(*cotE*) |  |
| CotE 525-6K-R | aagcttACTTTAAAAACTCCGGGTTG | | HindIII | +510/+526(*cotE*) |  |
| E-NdeI-F | TAGGcatatgTCTGAATACAGGGAAATT | | NdeI | +1/+21(*cotE*) |  |
| PDG364 720R-H3 | aagcttGGTAATGGTAGCGACCGG | | HindIII | +702/+720 (pDG364) |  |
| H34 | gagctcGATGAAGAATCAATCCAATTTACCG | | SacI | -1/+24 (*cotH*) |  |
| H35 | ggtaccTCATAAAATACTTAAATGATCTTTGAGG | | KpnI | +1062/+1086 (*cotH*) |  |

^a^ Capital and lowercase letters indicate nucleotides complementary to corresponding gene DNA and unpaired flanking sequences carrying a restriction site, respectively. ^b^ Underlined letters indicate codons which have been inserted. ^c^ Referred to *cotE* sequences, taking the first nucleotide of the initiation codon as +1.
